# A Simple Silver Nanowire Patterning Method Based on Poly(Ethylene Glycol) Photolithography and Its Application for Soft Electronics

**DOI:** 10.1038/s41598-017-02511-8

**Published:** 2017-05-23

**Authors:** Youngsang Ko, Jeonghun Kim, Dabum Kim, Yusuke Yamauchi, Jung Ho Kim, Jungmok You

**Affiliations:** 1 Department of Plant & Environmental New Resources, Kyung Hee University, 1732 Deogyeong-daero, Giheung-gu, Yongin-si, Gyeonggi-do 446-701 South Korea; 2 Institute for Superconducting and Electronic Materials (ISEM), Australian Institute for Innovative Materials (AIIM), University of Wollongong, North Wollongong, NSW 2500 Australia

## Abstract

Hydrogel-based flexible microelectrodes have garnered considerable attention recently for soft bioelectronic applications. We constructed silver nanowire (AgNW) micropatterns on various substrates, *via* a simple, cost-effective, and eco-friendly method without aggressive etching or lift-off processes. Polyethylene glycol (PEG) photolithography was employed to construct AgNW patterns with various shapes and sizes on the glass substrate. Based on a second hydrogel gelation process, AgNW patterns on glass substrate were directly transferred to the synthetic/natural hydrogel substrates. The resultant AgNW micropatterns on the hydrogel exhibited high conductivity (ca. 8.40 × 10^3^ S cm^−1^) with low sheet resistance (7.51 ± 1.11 Ω/sq), excellent bending durability (increases in resistance of only ~3 and ~13% after 40 and 160 bending cycles, respectively), and good stability in wet conditions (an increase in resistance of only ~6% after 4 h). Considering both biocompatibility of hydrogel and high conductivity of AgNWs, we anticipate that the AgNW micropatterned hydrogels described here will be particularly valuable as highly efficient and mechanically stable microelectrodes for the development of next-generation bioelectronic devices, especially for implantable biomedical devices.

## Introduction

Recent advancements in the fabrication of flexible and stretchable microelectrodes based on various conductive materials (i.e. graphenes, carbon nanotubes (CNTs), metals, conductive polymer, etc.)^1, 2^ and novel patterning techniques^3, 4^ have led to the development of novel bioelectronics^5^ such as wearable and implantable devices. In particular, hydrogel-based, molecularly-permeable microelectrodes are potentially valuable for soft bioelectronics applications. This is the case because hydrogels are well suited for interfacing with dynamic human tissues due to their biocompatibility, molecular permeability, and tunable mechanical properties^6–8^. Additionally, microelectrodes are amendable to rapidly detecting disorder in biological signals originating from various diseases^9, 10^. Despite the innovative utilization of hydrogel-based microelectrodes, there are fewer reports investigating hydrogel-based microelectrodes compared to glass- or silicone-based microelectrodes^11–13^. This discrepancy is related to the difficulty associated with constructing conductive patterns on wet hydrogel substrates.

Among various conductive materials, including conductive polymers^14–16^, carbonaceous materials (i.e. CNTs and graphenes)^17, 18^, and metal (Ag, Cu, Au etc.) nanomaterials^19–23^, the silver nanowires (AgNWs)^24–26^ show considerable promise for flexible microelectrodes due to their excellent electrical properties, flexibility, mechanical robustness, and low-cost processing^27^. Importantly, AgNWs have been demonstrated to exhibit an excellent long-term stability under ambient atmosphere, high temperature, and external force^28–30^. Because the performance of bioelectronic devices is highly dependent on the properties of electrodes consisting of the conductive materials, it is important to choose a highly conductive material. In general, carbon nanotubes, graphenes and conductive polymers are limited due to their low conductivity (100–1000 Ω/sq)^17, 31, 32^ and restricted processes. From these reasons, many researches have focused on the studies for patterning of metal nanowires. Jeong *et al*. reported reliable micropatterning method of AgNWs by the combination of lift-off process and spray coating process^33^. In addition, Ahn *et al*. reported a simple laminating process for the fabrication of AgNW patterns on paper substrate^34^. Recently, development of Cu nanowires (CuNWs) has been intensively carried out due to its low cost compared to AgNWs^35–40^. However, the coating process of CuNWs is very critical issue due to easy oxidation of Cu under air and wet conditions^23^. To utilize the CuNWs as an electrode material, therefore, further uses of an inert-gas or vacuum environment are required^41–44^. From these backgrounds, till now, solution-processable AgNWs under ambient condition can be considered as a proper candidate in conductive materials for the development of high-performance bioelectronic devices, especially, exposing to wet environments. Thus, the ability to pattern AgNWs on hydrogels is necessary in order to design new hydrogel-based microelectrodes with improved performance. Recently, Lee *et al*. reported a micropatterning technique for depositing AgNWs on a hydrogel substrate^11^. They constructed AgNW patterns on a glass substrate through a photolithographic process with a photoresist and then transferred the patterns to a hydrogel substrate *via* a direct transfer process. However, the photolithographic method is a time-consuming process that requires aggressive etching with toxic chemicals that can damage the electrical and mechanical properties of the AgNWs. More recently, our group demonstrated that a conductive polymer, organic conductor, i.e., poly(3,4-ethylenedioxythiophene) (PEDOT), can be successfully patterned on hydrogel substrates *via* only solution-based processes comprising solution phase monomer casting and oxidative polymerization and PEG photolithography^45^.

In this study, we successfully constructed highly conductive, flexible, AgNW-based microelectrodes on various substrates (i.e., glass, synthetic/natural hydrogels (PEG, polyacrylamide, and agarose), and polydimethylsiloxane (PDMS)) *via* a solution-based method. This method involves three steps: (1) AgNWs with various thicknesses and concentrations were deposited on a glass substrate by spin coating, (2) AgNW patterns were constructed on a glass substrate by poly(ethylene glycol) (PEG) photolithography, and (3) AgNW patterns on the glass were transferred to various flexible substrates, such as hydrogels, using a gelation process. After confirming the successful construction of AgNW patterns on both the glass and hydrogels, we investigated the electrical properties of the AgNW-based microelectrodes as a function of AgNW concentration and spin-coating speed. Our investigation revealed that these silver nanowire-based microelectrodes on hydrogels exhibited good electrical properties, excellent bending durability, and long-term stability in wet conditions.

## Results and Discussion

**Figure 1:**
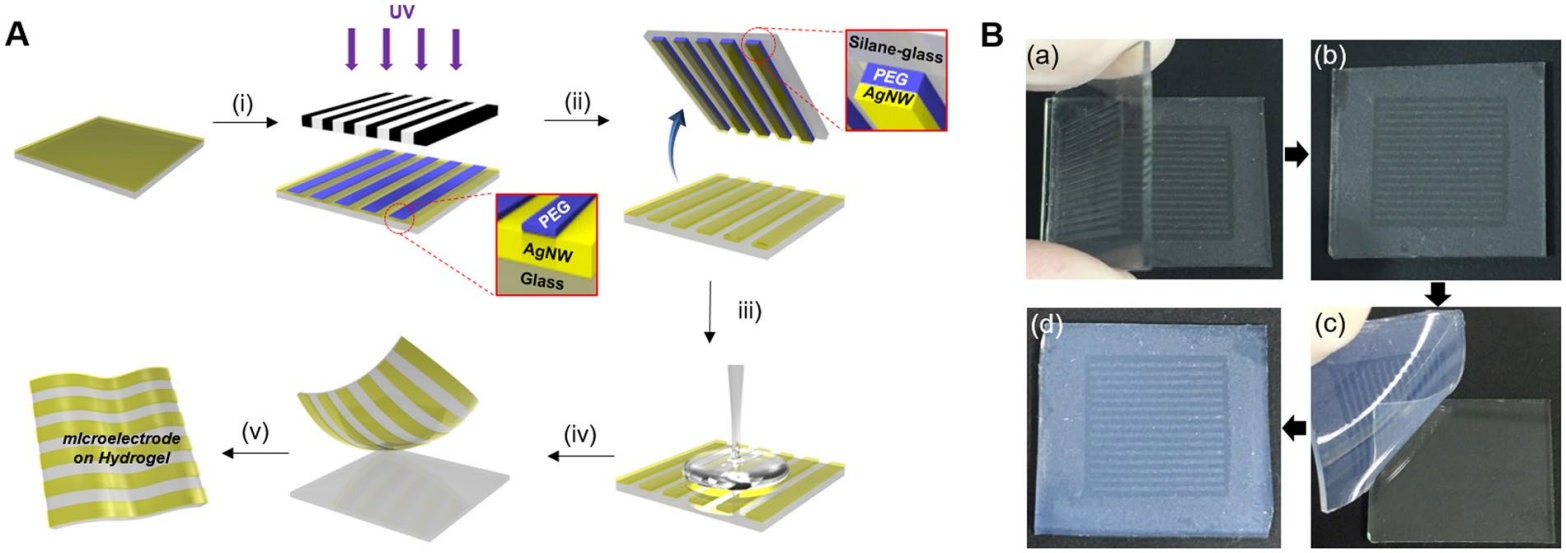
Fabrication of AgNW patterns on various substrates via two consecutive solution-only processes. (**A**) Schematic illustration for fabrication of AgNW-based micropatterns on the hydrogel: (i) AgNW dispersion solution was spin-coated on a glass substrate; (ii) PEG photolithography process using UV light via a photomask; (iii) peeling off of the PEG hydrogel layer by detaching the silane-treated cover glass to construct AgNW patterns on the glass substrate; (iv) second gel precursor solution (agarose or polyethylene glycol, or polyacrylamide) was poured onto the AgNW-patterned glass, and a second gelation process was conducted; and (v) peeling off of the second gel layer and transfer of the AgNW patterns from the glass to the hydrogel substrate. (**B**) Photographs showing the procedure used to fabricate AgNW-based micropatterns: (a) peeling off of the PEG hydrogel layer by detaching the silane-treated cover glass; (b) AgNW micropatterns on the glass substrate; (c) peeling off of the second gel layer; and (d) transfer of the AgNW patterns from the glass to the hydrogel substrate.

**Figure 2:**
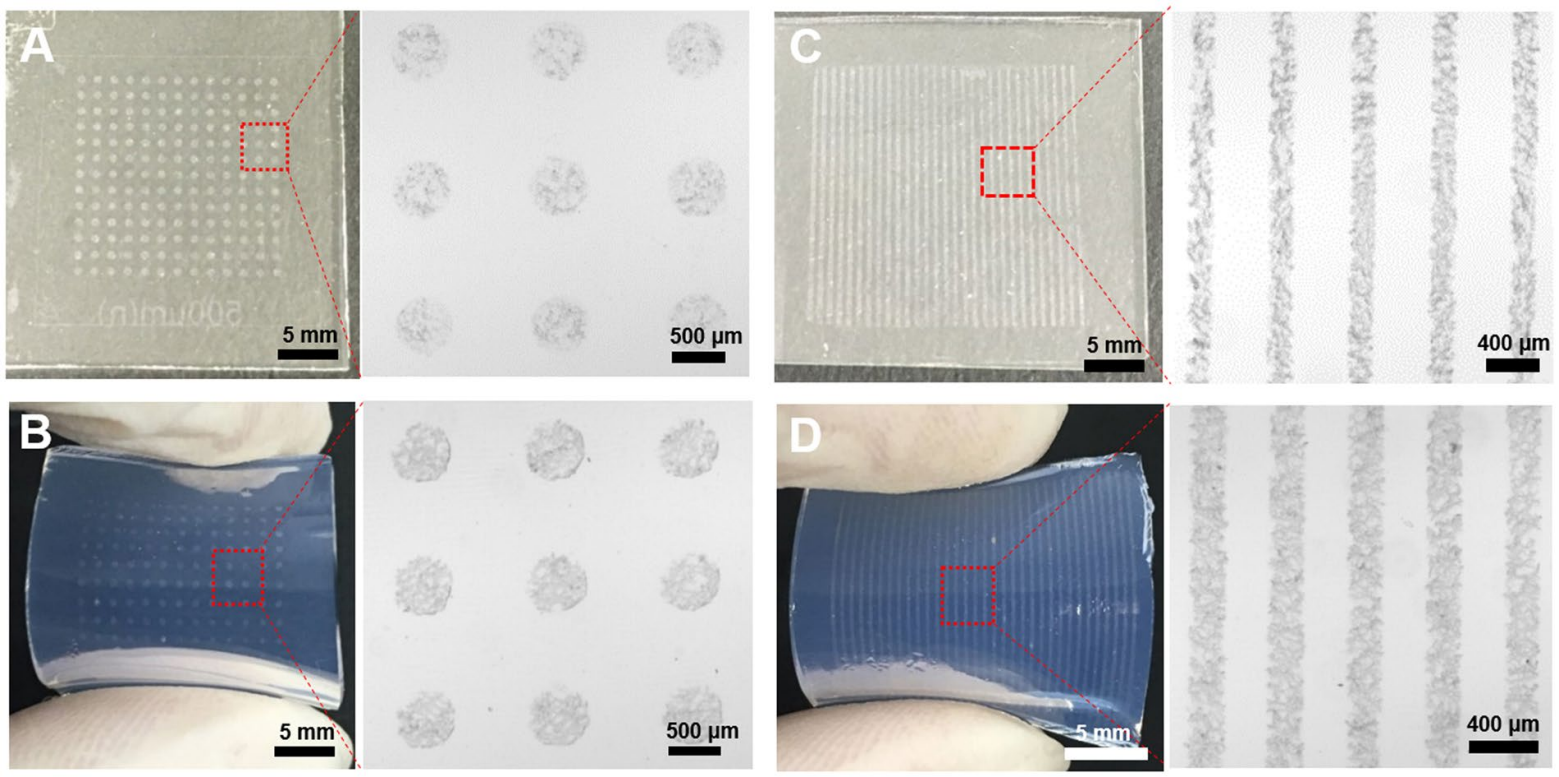
Photographic and optical microscopic images of AgNW-based micropatterns with (**A**,**B**) dot of 500 μm diameter and (**C**,**D**) line of 200 μm width) on the (**A**,**C**) glass and (**B**,**D**) hydrogel.

**Figure 3:**
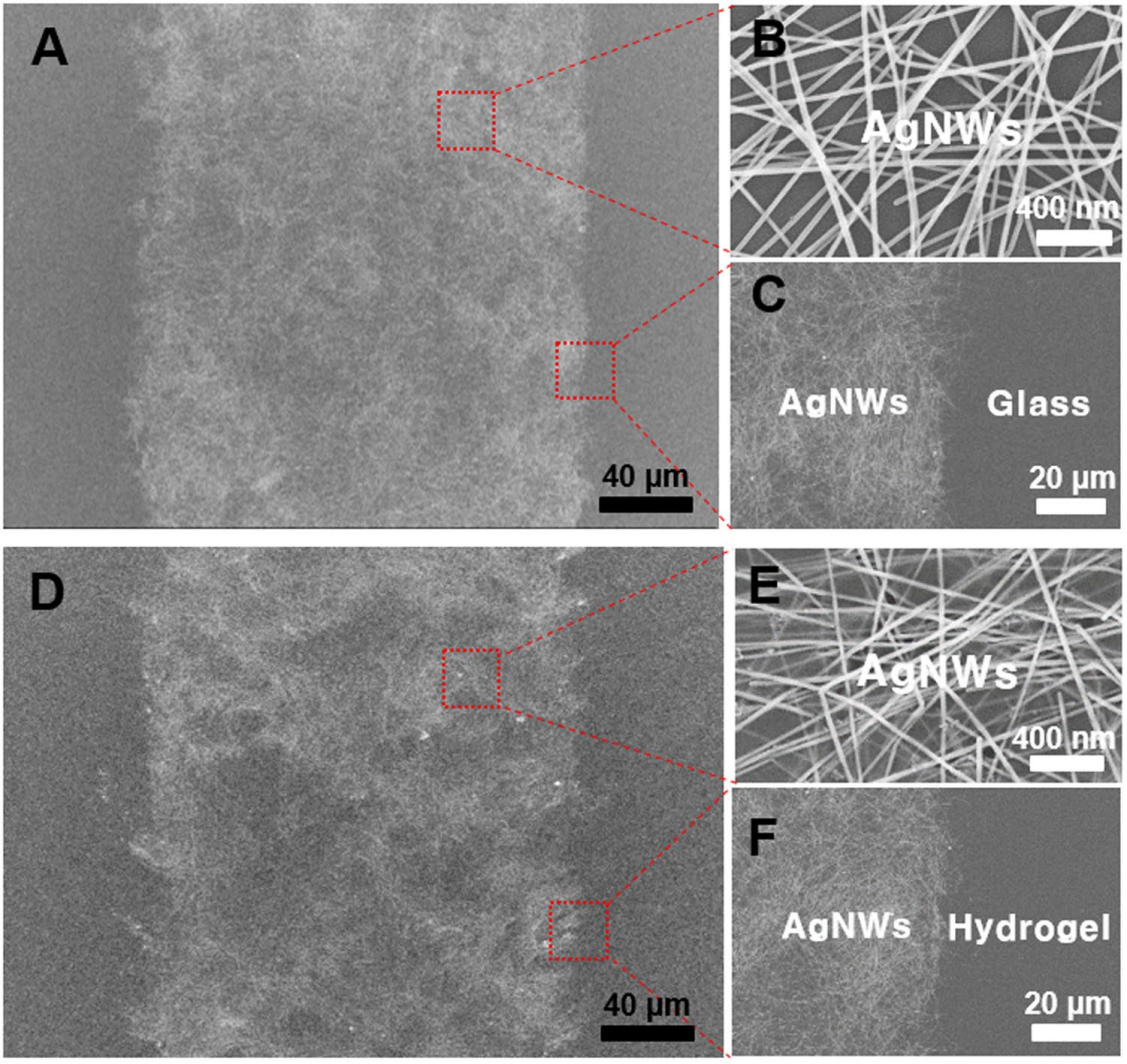
FE-SEM images of (**A**–**C**) AgNW-based micropatterns on a glass substrate before the pattern transfer process and (**D**–**F**) AgNW-based micropatterns on a hydrogel substrate after pattern transfer process. The line pattern width is 200 μm.

**Figure 4:**
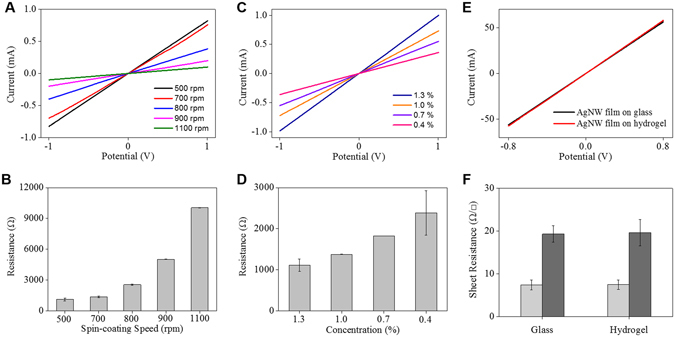
Electrical properties of AgNW-based micropatterns (700 μm in width and 2 cm in length) was measured by attachment of the copper tape on both sides. (**A**) Current-voltage (I–V) characteristics of AgNW-based micropatterns at the different spin-coating speeds and (**C**) various concentrations of AgNWs dispersion solution. Spin-coating speeds from 500 to 1100 rpm were used to control the thickness of the micropatterns and concentration from 0.4 to 1.3% were used to control the quantity of AgNWs. Resistance of AgNW-based micropatterns on glass substrate as a function of (**B**) spin-coating speeds and (**D**) concentrations. (**E**) Current-voltage (I–V) characteristics of AgNW-based micropatterns on glass before direct transfer, and hydrogel substrate after direct transfer process. (**F**) Sheet resistance of AgNW-based micropatterns on glass and hydrogel with the different spin-coating speeds.

**Figure 5:**
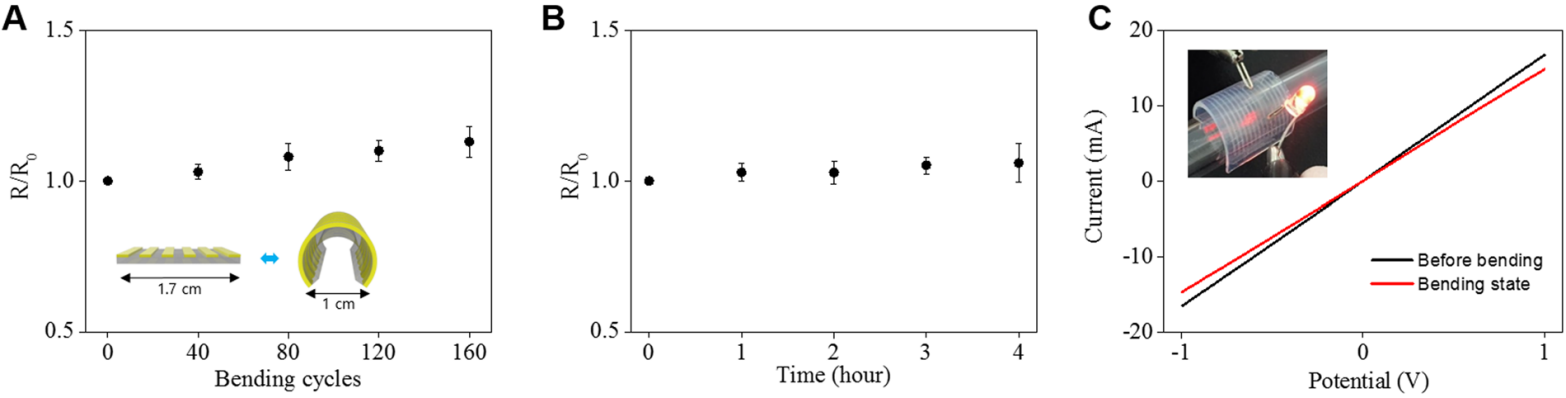
Mechanical flexibility or stability of AgNW-based micropatterns was measured by digital multi-tester device and compared to the initial value. (**A**) Bending test of AgNW-based micropatterns (700 μm in width and 2 cm in length) on PEG hydrogel. (**B**) Stability test of AgNW-based micropatterns on PEG hydrogel in DI water. (**C**) The I–V performance of the AgNW-micropatterns (500 μm in width, 2 cm in length) on hydrogel before bending and in bending state. The inset shows the LED emission with flexible AgNW-based micropatterns (500 μm in width and 2 cm in length) on agarose hydrogel.

**Figure 6:**
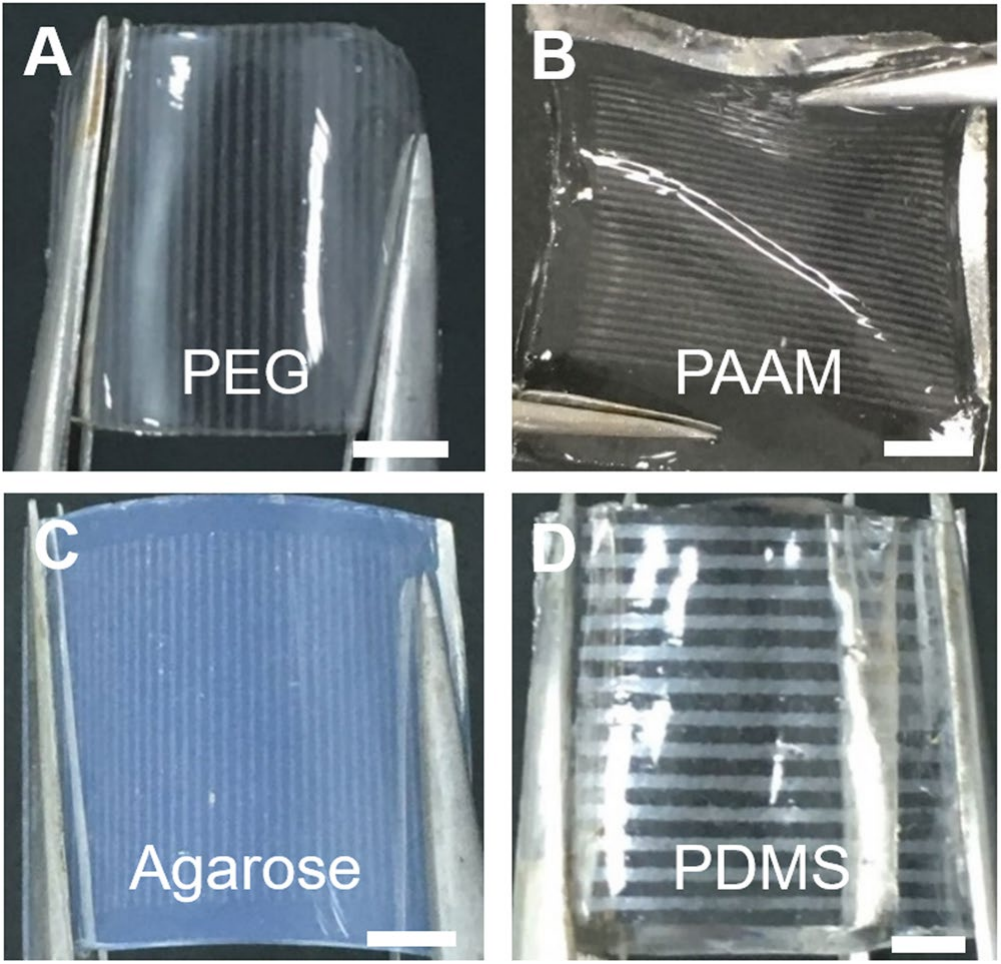
(**A**–**D**) Photograph images of AgNW-based micropatterns on various hydrogel or elastomer; (**A**) PEG (200 μm). (**B**) Polyacrylamide (PAAM) (200 μm) (**C**) Agarose (200 μm) and (**D**) PDMS (500 μm). Scale bar: 5 mm.

**Figure 7:**
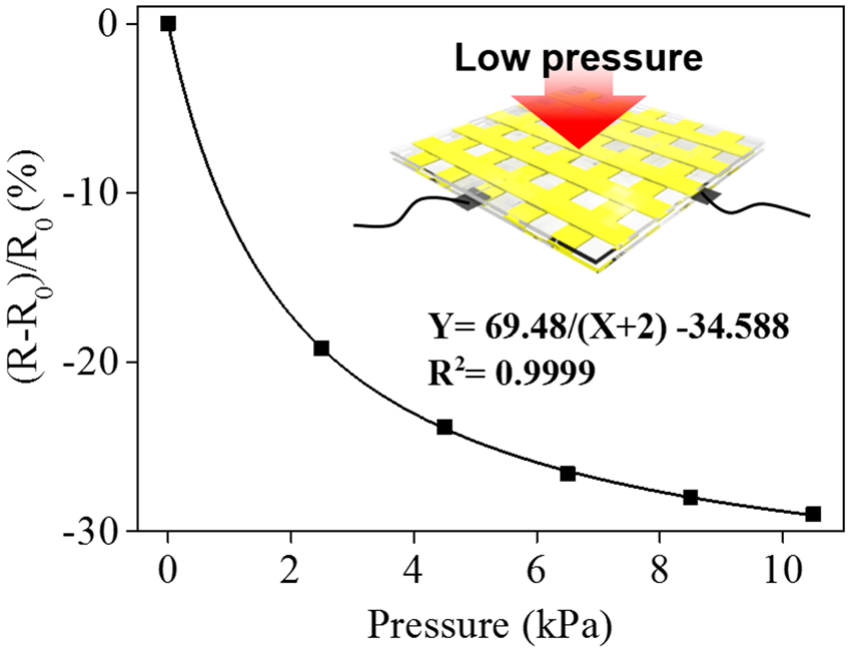
Fabrication of pressure-responsive device of two AgNW-microelectrodes on PEG hydrogel (1 mm on line pattern width) and tested the pressure response using hydrogel sandwich type. The inset shows that the experiments were conducted by applying pressure to the points where the line patterns with electrodes were engaged. The relative change of the resistance (RCR) graph of connected AgNW-microelectrode with different pressures (2.5, 4.5, 6.5, 8.5, and 10.5 kPa).

This study highlights how highly conductive AgNW materials can be micropatterned onto flexible substrates, such as natural/synthetic hydrogels, to construct hydrogel-based, biocompatible, molecularly-permeable microelectrodes. To accomplish this, we performed consecutive solution processes, as shown in the schematic illustration (Fig. 1A) and step-by-step photographic images (Fig. 1B). First, AgNW solutions of various concentrations (0.4, 0.7, 1.0, and 1.3%) were dropped and uniformly spin-coated onto a glass substrate at different spin-coating speeds (500, 700, 800, 900, and 1100 rpm) (Fig. 1A–i). Next, PEG photolithography was employed to construct AgNW patterns with various shapes (circles and lines) and sizes (500 μm and 200 μm) on a glass substrate (Fig. 2A,C). The PEG precursor solution was dropped onto the AgNW-coated glass, and then a silane-treated cover glass was placed over the PEG precursor solution. After UV-induced PEG gelation through the photomask, the PEG hydrogel was peeled off to detach the AgNWs from the glass substrate. Acrylate moieties present at the silane-treated cover glass were used to anchor the PEG hydrogel to the cover glass. The non-UV exposed AgNW region remained intact on the glass substrate, leading to the construction of AgNW patterns on the glass substrate. The AgNW detachment along with PEG hydrogel from the glass may be attributed to the strong adhesion between the PEG hydrogel and the AgNW film; this may originate from the strong cross-linking between polymer chains in the hydrogel and AgNW network structures (Fig. 1A–ii and B–a,b). In the final step, we directly transferred the micropatterned AgNWs from the glass to the hydrogel substrate *via* a second round of hydrogel gelation. In similar manner to the strong adhesion between the first PEG hydrogel and the AgNW film, a second synthetic (PEG, PAMM) or natural (agarose) hydrogel gelation process was employed to transfer the AgNW patterns from the glass to the biocompatible, flexible, molecularly-permeable hydrogel substrates (Fig. 1A–i–iii,iv,v and B–c,d). This strong adhesion between the AgNW network structures and hydrogels might lead to the successful AgNW pattern transfer from the glass to the hydrogel substrate. As seen from the photographic, optical microscopic images in Fig. 2, AgNW patterns with a dot width of 500 μm (Fig. 2A) and a line width of 200 μm (Fig. 2C) were successfully constructed on glass substrates *via* PEG photolithography. Furthermore, these AgNW patterns on the glass substrates were clearly transferred to hydrogel substrates by a transfer process based on the second gelation process (Fig. 2B,D). The SEM images presented in Fig. 3 revealed that the AgNWs patterns with line width of 200 μm were successfully constructed on the glass and hydrogel. Also, it means that the microelectrodes on the glass or the hydrogel are substantially no difference. Additionally, the resultant AgNW micropatterns can be completely transferred to hydrogel substrates without any damage to the AgNW patterns. Upon confirming the successful fabrication of AgNW patterns on both the glass and hydrogel substrates, we investigated the electrical properties of AgNW micropatterns as a function of spin-coating speeds and AgNW concentrations; these variables can determine the AgNW network density. We prepared AgNW-based micropatterns (700 μm in width and 2 cm in length) on the glass substrate using the PEG photolithography process described above and then measured I–V curves and electrical resistances with a two-point probe station. As shown in Fig. 4A and B, the electrical resistance of AgNW micropatterns increased from 1110 to 10 kΩ as the spin-coating speed increased from 500 to 1100 rpm, indicating that lower spin- coating speeds led to an increase in AgNW network density. Also, we demonstrated that the electrical resistance of AgNW micropatterns gradually increased from 1110 Ω to 2400 Ω as the AgNW concentration decreased from 1.3% to 0.4% (Fig. 4C,D). This suggests that higher AgNW concentrations in solution gave rise to higher AgNW network densities. These observations are consistent with previous reports indicating that denser networks of AgNW-based micropatterns induced improved electron transport pathways^11, 46^. We also evaluated the I–V characteristics and sheet resistances of AgNW-based micropatterns on the glass and hydrogel substrates before and after the pattern transfer process in order to investigate the effects of the pattern transfer process on the electrical properties of AgNWs. As seen in Fig. 4E and F, nearly identical I–V characteristics were observed for the AgNW-based micropatterns on the glass and hydrogel substrates. Moreover, the sheet resistances of AgNW micropatterns on the glass were determined to 7.4 Ω/sq and 19.4 Ω/sq at spin-coating speeds of 500 and 1100 rpm, respectively. The AgNW micropatterns on the PEG hydrogel had almost similar sheet resistances of 7.5 Ω/sq and 19.7 Ω/sq, respectively. These results strongly indicate that the pattern transfer process (from the glass to the hydrogel) does not have any detrimental effects on the electrical properties of the AgNW micropatterns. The AgNW patterns on the hydrogels described in this study exhibited good electrical properties comparable to previously reported values (8~13 Ω/sq)^47^. AgNW patterns on the hydrogel showed good electrical properties that were sufficient to turn on a light-emitting diode (LED) bulb (Fig. 5C). We further examined the mechanical flexibility of AgNW-based micropatterns on the hydrogel by analyzing the increase in electrical resistance during bending. In this test, AgNW-based micropatterns on the hydrogel underwent a bending strain of ~59%, with subsequent stretching; this was repeated for 160 cycles. As a result, a very small increase in the electrical resistance of the AgNW-patterned hydrogels was observed (around 3 and 13% after 40 and 160 bending cycles, respectively) (Fig. 5A). This indicates that significant cracking or fracturing of the AgNW network structures was not caused by repetitive bending cycles. The AgNW-patterned hydrogels were still electrically functional after 160 bending cycles. Importantly, Fig. 5C shows that AgNW micropatterns with bend radius of 6 mm remained functional to turn on a LED bulb. The I–V curve presented in Fig. 5C supported that AgNW micropatterns on hydrogel showed almost the same electrical property at bending state compared to that at non-bending state, indicating excellent bending durability as an electrode. In addition to the excellent bending durability of the AgNW-micropatterned hydrogel, these samples also exhibited good stability in wet conditions. As shown in Fig. 5B, the electrical resistance of the AgNW-micropatterned hydrogel only increased by ~6% after being immersed in water for 4 h. These flexibility and stability results verified that the highly conductive AgNW micropatterns on hydrogels can function as versatile, hydrogel-based microelectrodes for soft bioelectronics systems. The AgNW pattern transfer process described here is facile and well suited for various flexible substrates. As can be seen in the photographic images in Fig. 6A–D, the AgNW micropatterns prepared on the glass *via* PEG photolithography were successfully transferred to other hydrogels (e.g., polyacrylamide) as well as to elastomer substrates (e.g., PDMS). These results show that this method enables to apply into various bioelectronics applications using various gel species with different intrinsic properties. In comparison with previous studies^11, 33, 34, 48, 49^, our hydrogel-based patterning method has several advantages such as relatively inexpensive process, easily controllable process, and importantly, environmentally friendly process without toxic chemicals. This strategy described here required only common, inexpensive raw materials such as polyethylene glycol diacrylate (PEG-DA) and mild solutions such as water. In addition, this strategy can offer enough patterning resolution for microelectrode. As a proof of concept to demonstrate whether AgNW electrode on hydrogel could response to low stimulus environment, the pressure-responsive sensor was fabricated in form of sandwich-type composed of two AgNW electrodes with pattern width of 1 mm (Fig. 7). The relative change of the resistance (RCR) value ((R − R_0_)/R_0_) was calculated by resistance value obtained from the I–V graph at different pressures. It can be seen that the RCR value gradually decreased with pressure increasing from 0 to 10 kPa. It is important to note that AgNW patterns on hydrogel were highly responsive to low pressure sensing range (<2.5 kPa). It has been known that ideal blood pressure for a healthy adult is 16 kPa (120 mmHg) systolic and 11 kPa (80 mmHg) diastolic. Overall, the novel strategy described herein can enhance our ability to develop highly conductive, hydrogel-based microelectrodes for next-generation bioelectronics systems such as implantable blood pressure sensor.

## Conclusion

In this study, we developed highly conductive AgNW-based micropatterns on various substrates of glass, hydrogels (agarose, PEG-DA, polyacrylamide), and polydimethylsiloxane (PDMS) *via* solution-based processes without aggressive etching or lift-off processes. Using PEG photolithography, AgNW-based micropatterns of different shapes and dimensions were successfully constructed on glass. These AgNW patterns were cleanly transferred to synthetic/natural hydrogel substrates *via* a gelation-based transfer processes. The resultant AgNW micropatterns on the hydrogels exhibited high conductivity with low sheet resistance, excellent mechanical durability, and good stability in wet conditions. We anticipate that this simple micropatterning process will enhance our ability to develop next-generation bioelectronics systems as well as can be easily integrated into various soft electronics systems. Finally, we envisaged that AgNW-based microelectrode could be further improved in electrical conductivity by chemical welding process^50^ and patterning method described here could be applied to CuNWs for achieving the cost-effectiveness^51^ by new technologies, respectively.

## Experimental

### Chemical and materials

Silver nanowires (1.3 wt %) with 20–40 nm in diameter and 20–30 μm in length were purchased from NANOPYXIS. Poly(ethylene glycol) diacrylate (PEG-DA, MW 575); 3,4-acrylamide/bis-acrylamide solution (30%); ammonium persulfate (APS); and N,N,N′,N′-tetramethylethylenediamine (TEMED) were purchased from Aldrich Chemicals and used without further purification. 4-(2-Hydroxyethoxy) phenyl-(2-hydroxy-2-propyl) ketone (Irgacure 2959) was purchased from BASF. 3-Acryloxy-propyl trichlorosilane was obtained from Gelest, Inc. (Morrisville, PA). Agarose powder was purchased from Genomic Base. Toluene and ethanol were purchased from Duksan Pure Chemicals Company Co., Ltd. (Korea). Phosphate-buffered saline (PBS) was purchased from Life Technologies. The silicone elastomer base and silicone elastomer curing agent were purchased from Dowhitech Silicone Co., Ltd. (Goyang-si, Korea).

### Patterning of AgNWs on a glass substrate

The AgNW solution (1.3%, 1 ml) was dropped and spin-coated on a glass substrate at various speeds (500, 700, 800, 900, and 1100 rpm). To prepare AgNW films with various concentrations, AgNW solutions (1.3, 1.0, 0.7, and 0.4%) were dropped and spin-coated at 500 rpm. PEG-DA (MW 575) was mixed in PBS containing 1% w/v of a photoinitiator (Irgacure 2959), which was dissolved in 70% v/v ethanol, to achieve a 60% w/v gel precursor solution. The PEG precursor solution was dropped onto the AgNW-coated glass (2.5 × 2.5 cm^2^) and then covered with silane-treated glass. Silane modification was used to anchor the gel layer to the glass^52, 53^. Glass slides were treated with a corona surface treater to generate hydroxyl groups and then placed in a 2 mM solution of 3-acryloxy-propyl trichlorosilane in anhydrous toluene for 1 h. The reaction was performed in a nitrogen condition. After incubation, the glass slides were rinsed with fresh toluene, dried under nitrogen, and cured at 100 °C for 2 h. Silane-modified slides were placed in a desiccator until further use. The AgNW-coated glass was exposed to a UV light source (INNO Cure 2000, 2.32 mW cm^−2^) through a photomask for 1 s. The UV-exposed region of the AgNWs film was transferred to the silane-treated glass along with the PEG hydrogel. The region of the AgNW film that was not exposed to UV light remained intact to afford the AgNW patterns. The AgNW-patterned glass was dipped into ethanol to remove the unexposed PEG precursor solution. The average thickness of AgNW patterns was determined to be 159 nm from atomic force microscope (AFM) profile.

### Transferring the AgNW patterns from the glass to various substrates (PEG, agarose, PAAM, and PDMS)

The 3% w/w agarose gel precursor solution was heated to 90 °C to melt the agarose powder. The PEG precursor solution was used as described above. The polyacrylamide (PAAM) gel precursor solution was prepared by mixing distilled water (2.3 ml), 30% acrylamide/bis-acrylamide solution (5 ml), 10% APS solution (0.1 g APS + 1 ml distilled water, 100 μl), and TEMED (8 μl). The above hydrogel precursor solution (PEG, agarose, PAAM) was dropped onto the AgNW-micropatterned glass substrate. Gelation of the agarose was carried out by allowing it to cool at room temperature. Gelation of the PEG precursor solution was conducted by UV exposure. After gelation, the hydrogel was carefully peeled from the glass substrate. To transfer the AgNW patterns from the glass to the PDMS, the silicone elastomer base/silicone elastomer curing agent (20:1) composite liquid was prepared using a vacuum cylinder to eliminate air pockets in the composite liquid. The composite liquid was then poured onto the AgNW-micropatterned glass substrate. This was oven-dried for 1 h, and the PDMS was carefully peeled from the glass substrate. As a result, the AgNW micropatterns were cleanly transferred to various substrates without any defects.

### Measurement of pattern morphology and electrical properties

The morphologies of the AgNWs patterns were imaged *via* optical microscopy (Nikon eclipse Ti-S) and field emission scanning electron microscopy (FE-SEM, Hitachi, model S-4200, Carl Zeiss, model Merlin). To measure the resistance (Ω) of the AgNWs patterns, AgNW films were prepared on glass (2.5 × 2.5 cm^2^). After our patterning procedures, AgNW patterns (700 μm × 2 cm) were attached with copper tape on both sides. Measurements were taken using a two-point probe method from −1 V to 1 V with an electrical measurement device (PGSTAT204, Metrohm Autolab). In order to compare the resistance differences between AgNWs on glass and hydrogel substrates, AgNW films (2.5 × 1.5 cm^2^) on glass and hydrogel (after carrying out our patterning procedures) were measured by the two-point probe method from −0.8 V to 0.8 V. To measure the sheet resistance, AgNW-based micropatterns (500 μm in width) were prepared on the glass and hydrogel substrates. The measurement was performed by using sheet resistance tester (CMT-100S, Advanced Instrument Technology).

### Bending, stability, and LED bulb test

In the bending cycle test, the AgNW pattern (700 μm × 2 cm)-coated PEG hydrogel (2.5 × 2.5 cm^2^) was bent with a bending diameter of 10 mm and subsequently unbent; this was repeated for 160 cycles. The resistance of the sample was measured by a digital multi-tester device (HIOKI, model 3244–60) after 40, 80, 120, and 160 cycles; these values were compared to the initial resistance value. In the stability test, the AgNW pattern-coated PEG hydrogel was immersed in water for 4 h. The resistance of the sample was measured by a digital multi-tester device after 1, 2, 3, and 4 h and then compared to the initial value. For the LED bulb test, micropatterned AgNW films on PEG or agarose hydrogels were prepared. Electrodes were connected to the AgNW patterns through copper lines. A potential was applied *via* an electrochemical analysis device using chronoamperometry (potential: 2 V).

### Fabrication of pressure sensor device using AgNW-microelectrodes on hydrogel

In order to fabricate a pressure sensor device, two AgNW-microelectrodes with the pattern width of 1 mm were fabricated on PEG hydrogel and then assembled face to face with a small gap. The orientation of these two electrodes was perpendicular to each other. The I–V curves were obtained to demonstrate the pressure sensor at different external pressures. Finally, the relative change of the resistance (RCR) value ((R − R_0_)/R_0_) was calculated by the resistance value obtained from the I–V graph.
